# The Progress in the Diagnosis and Treatment of Affections of the Throat, Nose, and Ear during the past Twenty Years

**Published:** 1901-12

**Authors:** Barclay J. Baron

**Affiliations:** President, Physician in Charge of the Throat and Nose Department of the Bristol General Hospital.


The Bristol

Medico-Chirurgical Journal.

DECEMBER, 1901.

### THE PROGRESS IN THE DIAGNOSIS AND TREATMENT OF AFFECTIONS OF THE THROAT, NOSE, AND EAR DURING THE PAST TWENTY YEARS.

The Presidential Address, delivered on October 9th, 1901, at the Opening of the Twenty-eighth Session of the Bristol Medico-Chirurgical Society.

BY

Barclay J. Baron, M.B. Edin., President,

*Physician in Charge of the Throat and Nose Department of the Bristol General Hospital.*

I propose to put before you a few points in connection with the progress that has taken place in the diagnosis and treatment of disease of the throat, nose, and ear, in which I have been especially interested for the past twenty years. Before, however, proceeding to this my main subject, I may incidentally remark on the great increase in the facilities for studying these organs which has taken place during that time. At the beginning of this period, the British Medical Association did not devote a section to laryngology and otology; now this section is as important as any. Then the clinical teaching from patients affected with diseases of the throat, nose, and ear in the metropolis, and all large provincial towns, was mos rudimentary and imperfect. It was left to the general surgeons and physicians, who, in the rush of other work, were unable to devote much time to it. The laryngoscope was used by but few men in the country, and it was possible for a man to get his degree in any of our Universities, and never to have seen the vocal cords, to know nothing of the interior of the nose and its communicating sinuses, except anatomically. The existence of the lingual and naso-pharyngeal tonsils was not widely known, and the graduate might never have seen an operation for the removal of adenoid vegetations during his student days. I freely confess that this was my condition of crass ignorance when I entered the profession in 1881. I first saw the vocal cords, and watched with puzzled interest the insertion of Löwenberg's forceps behind the palate for the removal of adenoids, when a student in Schrötter's Clinique in Vienna in 1883. The accurate examination of the interior of the nose was quite impossible to me, and a considerable time elapsed before I was able to explore that *terra incognita.* It may be that I emerged from the teaching and examining mill less well prepared for the work before me than other men in this room who are my contemporaries, but I am inclined to believe that I was a fairly well-educated graduate. Men who have entered the profession during the past few years know how greatly this is altered. In London, special departments exist in all large general hospitals, several special hospitals and dispensaries have been established, and three flourishing societies which deal exclusively with these cases hold regular meetings. In every large provincial town in the country we find the same thing. Having had the opportunity of meeting in consultation a good many of my medical brethren, I know how increasingly keen is the interest which the profession at large takes in this subject.

During the time under our consideration, pathology, surgical methods, and therapeutics have made enormous strides. Bacteriological investigation, now so generally carried out, was practically born when Koch discovered the bacillus of tubercle in 1882, and we know how marvellously our knowledge of the causes of disease has been added to and made accurate by that discovery, and by the isolation of many other pathogenic organisms since that time. The whole domain of medicine and surgery has derived benefit from these discoveries, and in no portion of it is this more marked than in my special branch of professional work. Another discovery which has been of incalculable benefit to the laryngologist and rhinologist, without which, indeed, a large amount of our diagnostic and operative work could not be done, is that of cocaine in 1883. This has been followed by several other drugs of a kindred character, such as eucaine, orthoform, etc. More recently, we have used with much benefit—for the purpose of producing anæmia of the erectile tissues of the nose, and for preventing or stopping hemorrhage for diagnostic and operative purposes—extract of suprarenal capsule, a valuable representative of a class of drugs previously unknown to us.

Nowadays, the galvano-cautery has largely superseded all forms of mineral caustics, and, from its much greater controllability, is a safer and more efficient means of destroying redundant tissue, especially in narrow cavities such as the nostrils, glottis, etc. In his day, Morell Mackenzie was the most highly-educated rhinologist we had, but it is instructive to notice how very imperfect his knowledge of the nose and its cavities—as shown in his classical work, edition 1880—was as compared with ours. Just as charity is said to cover a multitude of sins, so catarrh was made to include many different affections of the nose, a prominent symptom of which was discharge. Little or no attempt was made to determine, by accurate observation, its character; still less was the exact point of its issue determined by examination of the interior of the nostril. "Purulent rhinitis" was the term often given to a fœtid purulent discharge coming from one or other of the sinuses. Great advance has been made in the systematic examination of the sinuses, and of their treatment when diseased. The antrum has been opened through the alveolus since John Hunter's day; but tapping it through the outer wall of the nostril by Krause's trochar, or through the canine fossa, or through both situations at the same time, is, comparatively speaking, recent, and is sometimes the only method of curing empyema with polypi, &c., in the cavity. The frontal sinus has been opened from the outside for many years past; but the rule laid down only a few years ago, that a very large opening must be made into the nose if perfect drainage and cessation of purulent discharge are to be obtained, must be looked upon as up-to-date frontal sinus surgery. The ethmoid cells we now recognise as a fertile ground for the production of pus and polypi, and we are constantly breaking them down for the cure of these troubles. The sphenoidal sinus which, not long ago, was declared by a well-known surgeon and anatomist, after experiments on the cadaver, to be unenterable by a probe, can, we now know, in certain cases, and especially if atrophic rhinitis be present, be explored and treated. This will, however, never be an easy operation, and in other than very expert hands may easily become a dangerous one. In helping us to come to a definite conclusion as to affections of the antrum and frontal sinus, we find trans-illumination by a Voltolini lamp, formerly not generally used, but now rarely omitted, of great value. My own experience of the result of operations on these sinuses for empyema tells me that success can by no means always be assured even when the operations are done by most experienced hands, but the earlier the mischief is recognised and the operation performed, the more likely are we to be successful.

Another thing that must strike all of us is the importance now attached to nasal respiration. Twenty years ago little or no attention was paid to this; now nearly everyone, professional and lay, is keenly alive to its importance. This naturally has led to the careful examination of the interior of the nose and naso-pharynx. We find that obstruction to nasal breathing occurs from deviation and spur of the nasal septum, hypertrophy of the middle and inferior turbinated bodies, polypi, cystic condition of the middle turbinates, of which I have had some notable examples in my own practice, hypertrophy of the posterior end of the inferior turbinates, the so-called "moriform"

growths, and adenoid vegetations in the naso-pharynx. The nasal saw and trephine are extensively used for the removal of bony growth, the spoke-shave for cartilaginous spurs and hypertrophied turbinates, and the galvano-cautery has largely displaced mineral caustics as formerly used. I have seen several instances of bridges between the septum and inferior turbinate caused by the careless use of caustics, and I never omit seeing a patient within a few days after the use of the galvano-cautery, to satisfy myself that I have not caused rawing of apposed surfaces which might lead to junction. For septal deviation, Asch's method of breaking the septum in attempting its replacement is a great advance on old methods. The latest operative procedure for the radical removal of nasal polypi consists in removing bone as well as soft tissue; and if we do not attempt this, we must thoroughly cauterise the stumps, or inevitably get the frequent recurrence of older days. It is very surprising to me to find how frequently "moriform" growths are unrecognised, presumably from too infrequent use of the posterior rhinoscopic mirror. Their removal by means of snare or guillotine gives splendid results where they are the cause of obstructed nasal breathing. If adenoids are not in everybody's mouth, the word is, and I shall not weary you by enumerating the many troublesome symptoms that arise from their presence. Fortunately, the man who denies their existence or who pretends he can get rid of them without operation is a *rara avis.* As well try to coax water out of a bottle without drawing the cork, as air through the nose and into the lungs of a person whose naso-pharynx is packed with adenoids without removing them. Seeing that in examining with the finger and in operating on them we are crushing tissues close to the mouth of the Eustachian tubes, it is not surprising that suppurative otitis is the penalty that the patient often pays from neglect of cleanliness and delicacy on the part of the operator. Small though this operation may be, care at the time, and afterwards, is extremely important.

The question of nasal reflexes, raised so prominently by Hack, has been much discussed during the time under review. Paroxysmal sneezing, sudden blocking up of the nostrils by swelling of the turbinated bodies, nasal hydrorrhœa followed by a short, dry, strangling cough, difficulty of breathing, and wheezing, are commonly observed reflex phenomena. Various irritants, from much-talked-of hay down to ordinary dust, alcohol, tobacco, etc., will set agoing this disagreeable train of symptoms in some people. It is interesting to note that salicylates have been strongly recommended in hay fever, and experience teaches that a gouty and rheumatic dyscrasia render a person more liable to trouble from irritable nasal mucous membrane than others. I have just lately seen a lady whom her medical man greatly benefited by administering salicylate of soda and morphia for the relief of extremely bad attacks of nasal irritability. I thoroughly cauterised both nostrils, and she is practically cured; and we must believe that whilst he treated her constitutional disabilities, I destroyed the sensibility of her nasal nerves, and this is certainly excellent collaboration. I cannot agree with Bosworth that a great proportion of cases of asthma are directly due to nasal abnormality, and can be cured by its rectification, because there are doubtless many causes of this affection. At the same time, I consider that the examination of an asthmatic patient is not complete without investigation of the interior of the nose, and over 50 per cent. of these patients will be found to have some unhealthy condition of its mucous membrane or bony and cartilaginous framework. Whilst it is not fair to promise cure, or even relief, of asthma from local treatment, at the same time we often obtain most satisfactory, and sometimes brilliant, results from it, and it is our duty to put the matter fairly before the patient. One of my own children suffered from asthma as badly as anyone I ever saw; I removed adenoids nine years ago, and she has never had an attack since the operation.

Our opinions have been greatly modified as regards the causation of inflammation, with thickening of tissue, granulations, &c., in the pharynx and larynx, because we now recognise the very important rôle that obstructive affections of the nose play in determining this, and that to cure a pharyngitis or laryngitis when chronic, we must usually get the nose right, if necessary by operation.

As regards the tonsils, we now have to recognise four—*i.e.*, two faucial, a naso-pharyngeal, and a lingual one. Within the past twenty years we have come to the conclusion that acute tonsillitis is frequently of rheumatic origin, and from other accompanying symptoms, this suggests the further idea of the pyæmic origin of both joint and tonsil inflammation. We find that the lingual tonsil is liable to the same affections as the others, although acute suppuration is, in my experience, extremely rare. On the other hand, chronic hypertrophy is quite common, and I am sure that many so-called cases of *globus hystericus* are, in reality, instances of this enlargement. The symptoms presented are somewhat alike, the patients are much more often women than men, are usually neurotic, and, unless one is alive to the possibility of affections of this gland setting up the symptoms we observe, injustice may be done to the patient and suitable treatment of an operative character delayed. In operations for its removal, especially where the galvano-cautery is used, great care must be taken not to injure the epiglottis, or serious and rapidly-occurring œdema may result, of which I have seen one case, happily not in my own practice.

Diphtheria is a disease which, in these later years, is much more accurately diagnosed and satisfactorily treated, both from the preventive and curative standpoint, than formerly. Owing to the systematic culture and examination of exudates, doubtless the number of cases notified to the Sanitary Authorities is greater than would have appeared in the days when the clinical symptoms alone were relied on. But allowing for milder cases, which might have escaped recognition, now coming under treatment, we yet are quite sure that our present-day therapeutic methods are much more successful than formerly. Not long ago I had a case of middle ear disease. During its progress the patient developed an exudation on the palate and uvula that looked like thrush. A swabbing was sent to the laboratory of our late distinguished president, Dr. D. S. Davies, and another was examined by Dr. Symes, both of whom found the bacilli of diphtheria. The exudate passed away in a day or two, and left no traces behind it; then it reappeared and again disappeared, and so on. It behaved, in fact, utterly unlike what one would expect a diphtheritic membrane to do, but the patient ultimately died after very slight exertion in her bedroom. Being puzzled as to the source of the infection, I made enquiry and found that the patient's son had had a sore throat some time before, of so trivial a character that he was not seen by a doctor, and she had nursed him. After her death I saw him for an abscess in the forehead—on opening which, necrosis of the frontal bone over the sinus was found to have taken place with communication into the nose. The boy told me that he had had a discharge of blood from the nostrils whilst his throat was sore, and I believe that he had diphtheria and infected his mother. I am quite sure that no one from seeing the mother's throat would have diagnosed diphtheria, bacteriological examination alone enabling us to do so.

From the *resumé* of Professor Sims Woodhead's "Report on the Bacteriological Diagnosis and the Antitoxic Serum Treatment, cases admitted to the Hospitals of the Metropolitan Asylums Board, 1895 and 1896," published in the *British Medical Journal*,[1] we read as follows:—

"RESULTS OF ANTITOXIN TREATMENT.

"Of the 2,503 cases under 5 years of age in which diphtheria bacilli were found, only 31.5 per cent. died, against 47.4 per cent. in the pre-antitoxin period. In 1895, in cases in which the fauces alone were affected, the percentage fatality was 12.1; when fauces and nares, 39.5; fauces and larynx, 37.3; larynx alone, 30.7; when all three, 62.2 per cent. In 1896, when the antitoxin treatment had improved, a considerable fall in the fatality occurred. In the pre-antitoxin year 1894 there were 261 tracheotomies, with a fatality of 70.4 per cent. In 1896, with 246 tracheotomies, the fatality was 41.4 per cent."

These statistics show that the free use of antitoxin does not increase the percentage of cases of albuminuria. Among the cases of paralysis following diphtheria, the fatality (32 per cent.) was higher among those not injected with antitoxin than among

[1] *Brit. M. J.*, 1896, i. 855, 926, 937.

those where antitoxin was used (30.5 per cent.), although the former "must be looked upon as being the result of comparatively mild attacks of the disease" not necessitating the administration of antitoxin. In 1896, when antitoxin was given much more freely, the fatality among the paralysed in the non-affected group fell to 18.4.

Without entering into further details on the figures, they clearly show the efficacy of the antitoxin when given at an early stage of the disease; and that the larger the quantity of antitoxin given during the earlier stages of the disease, the lower is the fatality. Conversely, the larger the number of cases that come under treatment in the later stages, however large be the quantity of antitoxin given, the higher is the percentage fatality.

The theoretical standard of 100 times the lethal dose has been taken as the amount necessary to neutralise one unit of antitoxin. Professor Woodhead found that in certain cases 20,000 units, and in one 40,000, of antitoxin were formed for every unit of toxin injected; while in no case in which there was any fever at all were there fewer than 63 antitoxin units for every unit of toxin injected. Animals treated with antitoxin, as pointed out by Roux and Vaillard in 1893, may lose blood equal to the whole body weight, if this loss be prolonged over a period of several days, without any material decline in the antitoxin value of the blood that remains. It is clear, therefore, that antitoxin still goes on being formed after all the toxin has disappeared, being probably "the continued result of a special stimulation of the antitoxin-forming cells."

When we come to the larynx, it is especially in our knowledge of laryngeal phthisis that we note an important advance. Previous to 1880 this disease, in all its stages, was regarded as absolutely incurable. In that year the feasibility of attacking it surgically was suggested. Heryng took this up very vigorously, and we began to think that we were, after all, dealing with a disease that was very amenable to treatment. Thus we had two periods—one of pessimism and one of optimism. Now we have reached a period when our opinion of the value of available treatment, after twenty years of trial, lies between these two extremes. We now say that during the inflammatory and infiltration stages, beyond enjoining strict rest to the voice and throat—*e.g.*, avoidance of speaking, inhalation of irritating vapours or dust as in manufacturing or distributing trades, the taking of irritating articles of food or drink, tobacco-smoking, &c.,—we can do little by local treatment to stay the progress of the disease. When, however, we have to do with dysphagia our armamentarium has been enormously enriched by the discovery of cocaine, eucaine, &c. Surgery in these early stages of the disease is as yet only at the beginning of its trial. In the third or ulcerative stage we are now able to say that we can occasionally, but unhappily rarely, show a case in which local, combined with modern sanatorium treatment has brought about healing of tuberculous ulceration. But we are not entitled, I think, to speak of cure until at least one year has elapsed without recurrence. We also know that the only condition of the patient in which we are at all likely to obtain this much-to-be-desired result is that in which we get a healing fibroid state of the lungs established, but not always then. There is no room for optimism in this; but yet we have advanced far from the position held previous to 1880, and this advance is mainly due to our having attacked the diseased structures surgically. When, however, we think of the power of alleviating the awful suffering to which some of these patients are subjected, which the discovery of cocaine and eucaine, and, in the ulcerated stage more especially, orthoform, has placed in our hands, we cannot but acknowledge our great indebtedness to the chemist and therapeutist for their marvellous discoveries. Very recently I have seen a case in which insufflations of orthoform and resorcin apparently completely cured undoubted laryngeal tubercular ulceration, and this simple method of treatment is worthy of further trial.

During the past twenty years a large number of operations for the removal, entire or partial, of the larynx have been undertaken for the extermination of cancer. Powers and White[1] give the following table:—

[1] *Med. Rec.*, 1895, xlvii. 353; quoted by Jonathan Wright in *An American Text-Book of Diseases of the Eye, Nose and Throat.* Edited by de Schweinitz and Randall. Vol. ii., 1899, p. 1112.

| | | |
|---|---|---|
| Total excision ... ... ... ... ... ... | 180 | cases. |
| Died as result of operation ... ... | 72 | ,, |
| Died in first year ... ... ... ... | 8 | ,, |
| Recurred in first year ... ... ... | 51 | ,, |
| Recurred after one year ... ... ... | 8 | ,, |
| Reported free in first year ... ... | 16 | ,, |
| ,, in second year... ... | 11 | ,, |
| ,, in third year ... ... | 3 | ,, |
| ,, after three years ... | 11 | ,, |
| Partial excision ... ... ... ... ... ... | 77 | ,, |
| Died as result of operation ... ... | 26 | ,, |
| Died in first three years ... ... ... | 5 | ,, |
| Recurred in first year ... ... ... | 17 | ,, |
| ,, after first year ... ... ... | 3 | ,, |
| Reported free in first year ... ... | 13 | ,, |
| ,, in second year... ... | 4 | ,, |
| ,, in third year ... ... | 2 | ,, |
| ,, after three years ... | 7 | ,, |

These statistics show us that only a small proportion of cases operated upon are cured. Doubtless better results will be obtained when every one of us is in the habit of examining every case of hoarseness, however slight, with a laryngoscope. This symptom is for some time usually the only one present in laryngeal carcinoma, and if we find swelling about the arytenoid with interference of movement of the attached cord during quiet respiration in a patient otherwise healthy, and especially in middle life, apart from the discovery of suspicious growth within or outside the larynx, this should arouse our suspicions. I am quite sure that from omission of laryngoscopic examination the diagnosis is often made much later than need be, and the results of operation will become more satisfactory when diagnosis is made and operation is done earlier, rather than from improvement in surgical technique. There are, apparently, cases on record of successful removal of malignant growth by endo-laryngeal methods, but we can scarcely believe that an infiltrating growth can often be successfully dealt with in this way. The question of the risk which a patient runs where we operate on benign laryngeal growths, *per vias naturales*, of a conversion into epithelioma has been settled by Semon's statistics. These make it 1 in 1645, and even here it is difficult to say whether some of these growths, apparently innocent, were not really always malignant. Several years ago I published a case with Semon that appeared to justify us in holding the opinion that this transference can occur.

Reviewing our present knowledge of the motor innervation of the larynx, we know that the crico-thyroid muscle receives motor impulses from the exterior branch of the superior laryngeal nerve, and, as shown by Exner, Onodi, and others, from the middle laryngeal communicating branch from the pharyngeal branch of the pneumogastric. All other intrinsic laryngeal muscles derive their motor nerve supplies from the recurrent laryngeal. In 1881 attention was called to the fact that in laryngeal paralysis the abductors are the first, and sometimes, apparently, the only muscles that suffer as a result of central or nerve trunk lesion. In 1884 Krause considered that his experiments on animals enabled him to say that this apparent paralysis is due to contracture of all the laryngeal muscles from irritation caused by pressure on the nerve trunks or centres, or by their involvement in disease. Various other theories have been launched; *e.g.*, Mackenzie and Riegel suggested the existence of separate filaments in the recurrent supplying the different muscles, and Onodi and Risien Russell demonstrated their existence. Hooper showed that ether has a peripheral and differential effect upon the laryngeal muscles, the abductors being the first to suffer in their functions. Jeanselme and Lermoyez showed that they lose their contractility first after death. Fränkel and Gad showed that freezing the recurrent affects them first, and Massini that chromic acid applied to the nerve does the same. Grabower has adduced evidence that the greater vulnerability may be due to a difference in the method of termination of the nerve fibres in the muscles. Semon, who, in collaboration with Victor Horsley, did so much to endeavour to elucidate this matter, thinks that the muscles themselves may have different characteristics. In our Society we have members who are greatly distinguished in the physiology and pathology of brain and nerve, and I would venture to suggest that they may add further lustre to their fame by investigating this subject.

Paralysis of larnygeal muscles has been observed in many lesions of brain and spinal cord. The most interesting is tabes dorsalis: of 122 cases observed in Gerhardt's clinique, paralysis of laryngeal muscles was seen in seventeen. In eleven of these the abductor muscle was affected: in five bi-laterally, in four on the right side, and in two on the left. There were three cases of paralysis of all the muscles supplied by the recurrent—one bi-lateral and two on the right side. Of the three cases, there was one each of paralysis of the recurrent and of the superior laryngeal, and once paralysis of the recurrent of one side and of the abductor muscle alone of the other. In two cases paralysis of the thyro-arytenoid was seen; ataxic movements of the cords were seen in two cases, and laryngeal crises in four.

The discovery of Röntgen rays has greatly helped the laryngologist in accurately localising the cause of recurrent nerve paralysis. Such a case as I recently reported at a meeting of this Society, and which is published in the last number of this *Journal*, is most instructive in this respect. I would like to strongly urge that whenever we see a case of immobility of a vocal cord, partial or complete, with no pathological appearance in the larynx nor affection of central origin to account for it, we ought never to omit to call in the skiagraphist. The case I have published shows how large an aneurysm may be present without giving rise to symptoms other than laryngeal. Quite recently, on the other hand, I have seen a case of gôitre which has been in existence for the past two years, has grown considerably during the last six months, and in which the symptom of hoarseness, due to paralysis of the left vocal cord, has manifested itself during the past three weeks. On examination with the fluorescent screen, the thorax was found to be quite healthy, and the pressure on the nerve by the enlarged thyroid could be said, with certainty, to be the cause of the paralysis.

A few years ago a well-known general surgeon told an equally well-known aurist that a lecture to students on deafness could rightly be made very short. In was in these terms:—"There are three causes of deafness and three methods of treatment: 1. Wax in the ear; squirt water into it, out it comes—result, cure. 2. Blockage of Eustachian tube; stick a tube up the nose, patient gulps, bang goes the bag—result, cure. 3. Nerves; nothing to be done."

Nowadays, with graduated tuning forks, Galton's whistle, and accurate application of Weber's and Rinne's tests, we classify our cases with considerable accuracy. The respect paid to a discharge of pus from the ear by most of our profession is vastly greater to-day than it was twenty years ago. Then the dangerous old saying: "The child will grow out of it—let it alone," was very commonly heard, and extensive removal of the membrana tympani and the ossicles, the formation of polypi, permanent deafness, invasion of the mastoid and brain, were the results of the preaching of this pernicious doctrine. Surgeons who were thoroughly convinced of the necessity of scrupulous cleanliness and free drainage in the treatment of an abscess in a limb, allowed the middle ear to be filled with stinking pus, which percolated through a small hole in the drum, frequently placed in the worst possible position for drainage, and did nothing for its relief. Even now there is great room for improvement in the amount of care bestowed on cases of suppurative otitis, and it cannot be impressed too strongly on the profession at large that it is the bounden duty of every man who treats a case of this kind to do everything in his power to make the middle ear aseptic and establish free drainage. The best results are obtainable by accurate early treatment in an acute case, because we shall then usually succeed in preventing permanent perforation and secure perfect hearing. In order to do this, experience teaches us that we must deal not only with the ear, but with the nose and naso-pharynx. As a rule, inflammation and the formation of middle ear abscess originate in the nose, naso-pharynx, and Eustachian tube. We must, therefore, see to it that we restore normal health to these passages and cavities. It is a matter of common experience that frequently the only way to heal a purulent discharge from the middle ear is to remove abnormal structures and growths from the nostril, and adenoids from the naso-pharynx. No one can thoroughly treat a case of suppurative otitis who does not make it a matter of routine practice to examine and, if necessary, treat the nose and naso-pharynx The same remark applies to the treatment of Eustachian tube obstructions. All who have had any experience in the treatment of these cases know how frequently we fail to keep the tube open by passing a catheter or by using Politzer's bag. In many such cases we shall secure excellent results by removing all nasal and naso-pharyngeal abnormality, and thus secure permanent ventilation of the middle ear.

The modern operation for freely entering and draining the mastoid antrum, with skin grafting of the wound track, marks a great advance in this class of surgery, as also does the removal of the ossicles in certain forms of chronic suppuration. I will not weary you furthur, but hope I have succeeded in interesting you with the relation of my own experience of twenty years of professional work.

